# Extracellular Superoxide Dismutase, the Endothelial Basement Membrane, and the WNT Pathway: New Players in Vascular Normalization and Tumor Infiltration by T-Cells

**DOI:** 10.3389/fimmu.2020.579552

**Published:** 2020-10-30

**Authors:** Diego Martínez-Rey, Lorena Carmona-Rodríguez, María Jesús Fernández-Aceñero, Emilia Mira, Santos Mañes

**Affiliations:** ^1^ Department of Immunology and Oncology, Centro Nacional de Biotecnología (CNB/CSIC), Madrid, Spain; ^2^ Proteomics Unit, Centro Nacional de Biotecnología (CNB/CSIC), Madrid, Spain; ^3^ Department of Surgical Pathology, Fundación de Investigación Hospital General Universitario Gregorio Marañón, Madrid, Spain

**Keywords:** oxidative stress, ROS, laminin, diapedesis, extracellular matrix, FoxM1, immunotherapy, β-catenin

## Abstract

Tumor-infiltrating lymphocytes (TILs) are major players in the immune-mediated control of cancer and the response to immunotherapy. In primary cancers, however, TILs are commonly absent, suggesting T-cell entry into the tumor microenvironment (TME) to be selectively restricted. Blood and lymph vessels are the first barriers that circulating T-cells must cross to reach the tumor parenchyma. Certainly, the crossing of the endothelial cell (EC) basement membrane (EC-BM)—an extracellular matrix underlying EC—is a limiting step in T-cell diapedesis. This review highlights new data suggesting the antioxidant enzyme superoxide dismutase-3 (SOD3) to be a regulator of EC-BM composition in the tumor vasculature. In the EC, SOD3 induces vascular normalization and endows the EC-BM with the capacity for the extravasation of effector T-cells into the TME, which it achieves *via* the WNT signaling pathway. However, when activated in tumor cells, this same pathway is reported to exclude TILs. SOD3 also regulates TIL density in primary human colorectal cancers (CRC), thus affecting the relapse rate and patient survival.

## Introduction

The neoantigens expressed by cancer cells and the oncogenesis-associated inflammation might induce specific and potent antitumor immune responses. However, cancers develop mechanisms that help them avoid immune-mediated suppression (1). One involves the exclusion of tumor-specific T-cells from the tumor parenchyma, giving rise to so-called “cold” tumors. The chemoattractant milieu induced by tumor cells is restrictive towards tumor-infiltrating lymphocytes (TILs), preventing their diapedesis while supporting the chemotaxis of cells that suppress TIL function (2). Molecular and physical alterations of the tumor-associated endothelium can also influence T-cell extravasation (3). Indeed, the correction of vascular abnormalization with anti-angiogenics can have a synergistic effect with immunostimulatory drugs (including immune checkpoint inhibitors) and improve disease control and overall survival in different types of cancer (3–5). The identification of processes and molecules able to normalize tumor vasculature might therefore offer a means of improving cancer immunotherapy.

Vascular dysfunction in many pathologies including cancer, is closely related to the aberrant accumulation of reactive oxygen species (ROS) in perivascular areas (6). ROS affect structural and cellular components of the vascular wall, including the endothelial cell (EC) and vascular smooth muscle cells (VSMC) (7). Oxidative stress is caused by an imbalance between ROS production and antioxidant defenses (8, 9), and ROS accumulation induces cell and tissue damage *via* the direct oxidative modification or annihilation of lipids, proteins, and nucleic acids. Damage also occurs indirectly through nitric oxide (NO) oxidation and the formation of reactive nitrogen species (RNS) (10). In addition, oxidized and S-nitrosylated molecules act as second messengers in many signaling pathways (11). Thus, it is not surprising that complex enzyme networks for maintaining ROS homeostasis should exist (12). Key enzymes in such networks include the superoxide dismutases (SODs), which catalyze the dismutation of the superoxide anion ($\cdot {O_{2}}^{-}$) into molecular oxygen (O_2_) and hydrogen peroxide (H_2_O_2_). In mammals there are three SOD isoforms that differ in cell compartmentalization and the metal cofactor they require: SOD1 is cytosolic, SOD2 mitochondrial, and superoxide dismutase-3 (SOD3) extracellular, and while SOD1 and SOD3 use copper and zinc as cofactors, SOD2 uses manganese (8, 9).

The present review focuses on SOD3 since two recent reports have shown that the cancer-driven repression of this anti-oxidant enzyme is related to tumor vessel abnormalization and T-cell exclusion in both experimental and primary colorectal cancers (13, 14). The restoration of SOD3 levels corrects vascular function and structure, and induces the specific extravasation of effector T-cells. Both processes involve the activation of a hypoxia-inducible factor (HIF)-2α transcriptional program that remodels EC cell-cell contacts and renders the EC-basement membrane (BM) permissive towards T-cell diapedesis.

## Vascular Barriers to T-Cell Extravasation

During extravasation into tissues or tumors, circulating leukocytes must cross at least two barriers. The first is that formed by the EC. Crossing this layer requires a coordinated sequence of adhesive interactions between different diapedesis receptors, integrins, and chemokine receptors expressed by the leukocyte and the EC. Among these receptors are the E- and P-selectins, platelet endothelial cell adhesion molecule 1 (PECAM1), ICAM (intercellular adhesion molecule)-1/2, and VCAM (vascular cell adhesion molecule)-1 [extensively reviewed in (15)]. These adhesion molecules induce the arrest of and morphological changes in leukocytes that allow diapedesis. Diapedesis can occur in-between EC (paracellular) or directly through the EC body (transcellular). The stability of the EC barrier plays a major role in the diapedesis route chosen by leukocytes (16). An important molecule in paracellular diapedesis is vascular-endothelial cadherin (VEC; CD144, CDH5) a component of adherens junctions (AJ). VEC acts as a barrier to extravasation by establishing trans-homophilic interactions with other VEC molecules, and by anchoring these complexes to the cytoskeleton *via* their interaction with cytosolic β-catenin and p120-catenin (17). VEC release from p120 and β-catenin is tightly regulated by a complex network of phosphatases, kinases, and AJ components, eventually allowing leukocyte extravasation (18). The receptors required to cross the EC layer are common to all leukocyte subtypes (15).

**Figure 1:**
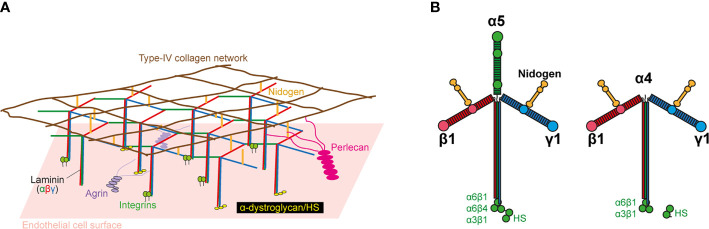
Basic structure of laminin and the endothelial cell basement membrane (EC-BM). **(A)** Molecular structure of EC-BM. The laminin network anchors to ECs by interacting with integrins, a-dystroglycan, and sulfated glycolipids. Lateral interactions are made with agrin and perlecan. The laminin network links to an independent type IV collagen network *via* nidogen and HS. **(B)** Lm511 and Lm411 heterotrimers. Domain locations for laminin-binding to nidogen and cellular receptors are indicated.

The second barrier to extravasation is that formed by the EC-BM, a 50–100 nm-thick, sheet-like structure underlying ECs (19). It is composed of a mixture of elastin, collagen IV, enactin/nidogen, heparan-sulfate proteoglycans, and laminins (**Figure 1A**), which together form a complex three-dimensional topography of pores and fibers. The EC-BM influences the shape of the EC, their migration, differentiation, and proliferation (20, 21), as well as diapedesis in leukocytes (22, 23). Most of the effects of the EC-BM on EC function and leukocyte trafficking are mediated by laminins, a family of secreted multidomain heterotrimeric proteins composed of α, β, and γ subunits. There are five α, four β, and three γ genes in humans, and their products can be arranged into 16 different T-shaped trimers (**Figure 1B**). Whereas the β and γ chains are ubiquitous, the α chain is restricted to certain tissues and cell types. The two predominant isoforms in the EC-BM are laminin 411 (α4β1γ1; Lm411), which is present in all blood vessels, and Lm511 (α5β1γ1), which is expressed in capillaries and venules (24). Laminin α4 (LAMA4) deficiency in mice results in capillary bleeding and hemorrhages; larger vessels are unaffected (25). Deletion of the laminin α5 gene (LAMA5) in mice results in embryonic lethality at E10-11, despite local compensation by other laminins in certain organs (26, 27). However, EC-specific LAMA5-deficient mice are viable and show no defects in blood vessel formation (28). This may be due to a counterbalance in the expression of LAMA4 and LAMA5. Certainly, the embryonic bleeding and perinatal anemia observed in LAMA4-deficient mice is rescued upon postnatal LAMA5 expression, which would help stabilize the vascular system. This suggests that neither LAMA4 nor LAMA5 are essential for vasculogenesis.

## Role of Laminins in T-Cell Diapedesis

Laminin α chains not only affect EC function in different ways, but also have contrasting effects on leukocyte diapedesis. Initial evidence suggested that LAMA4-containing laminins provide specific permissive signals for transendothelial T-cell migration, whereas those containing LAMA5 inhibit T-cell transmigration (29). Inflammatory mouse models indicate that T-cells, neutrophils, and monocytes extravasate preferentially at LAMA5-low sites (25, 30–32). The patchy distribution of LAMA5 *vs* the ubiquitous expression of LAMA4 in capillaries might create EC-BM regions with different potentials for leukocyte extravasation (31). LAMA5 patchiness does not, however, correlate with pericyte coverage. It is curious that T-cells adhere more strongly to LAMA5- than to LAMA4-containing laminins, but migration is faster across those with LAMA4 (33). LAMA5 might inhibit leukocyte diapedesis *via* adhesion-independent mechanisms. Indeed, LAMA5 induces VEC redistribution and stabilization at cell-cell junctions (34), which hampers paracellular diapedesis.

LAMA4 seems not to play a direct role in specific T-cell diapedesis, but the LAMA4/LAMA5 balance is critical in T-cell-mediated immune responses. Regulatory T-cell (T_REG_) trafficking into lymph nodes is a requisite for suppression of alloantigen-specific CD4^+^ T-cell priming (35). The LAMA4/LAMA5 ratio in the EC-BM of high endothelial venules (HEV) is a major determinant of whether lymph nodes are tolerant to an antigen (high LAMA4) or respond to it (high LAMA5) (30). LAMA5 overexpression prevents the transmigration of T-cells and dendritic cells (DC) through lymph nodes, whereas LAMA4 silencing bypasses the induction of tolerance and triggers allograft rejection. Thus, the LAMA4/LAMA5 balance regulates antigen tolerance or immunity by regulating T-cell diapedesis across HEV EC.

The role of laminins in immune responses is not limited to the control of trafficking. Lm511 increases phagocytosis *via* monocyte-derived DCs, leading to antigen-presenting cells with stronger T-cell stimulating capacity (36). Laminins have been reported to directly affect T-cell activation and polarization, although this is not free of controversy. In one study, Lm411 and Lm511 induced CD4 T-cell co-stimulation (37), and in another Lm411 and Lm511 had opposite effects on lymph node-derived CD4 T-cell activation and proliferation (38). The latter study also reported Lm511 to enhance CD4 T-cell polarization towards the T-helper (Th)1 and Th17 phenotypes, whereas Lm411 biased CD4 T-cells towards T_REG_ cell differentiation. However, using encephalitogenic CD4 T-cells, Sorokin’s group showed an inhibitory role for Lm511 in Th1 and Th17 CD4 T-cell polarization (33).

## Changes in The Endothelial Cell Basement Membrane Associated With Tumor Progression

The EC-BM of the tumor vasculature is morphologically abnormal, a result of the continuous remodeling associated with endothelial sprouting and vessel growth (39). Cancer- or EC-induced EC-BM remodeling is also a major driver of tumor angiogenesis (40). Fully assembled EC-BM usually provide growth arrest cues that maintain EC barrier function. EC-BM degradation, however, triggers angiogenesis by i) fostering the loss of pericytes from EC, ii) releasing extracellular matrix-sequestered pro-angiogenic factors, and iii) exposing the cryptic domains of partially degraded collagens with pro-angiogenic activity (41–43). Curiously, EC-BM degradation also generates ECM fragments with anti-angiogenic activity (44, 45).

The composition of the EC-BM is strongly influenced by the inflammatory milieu in the tumor microenvironment (TME). For example, Lm111 is modified by ectokinases secreted by immune cells during extravasation, which alters the properties of the EC-BM barrier (46). In breast carcinomas, pro-inflammatory cytokines, such as tumor necrosis factor (TNF)-α, suppress laminin α3B (LAMA3B) but enhances LAMA4 expression (47). In the breast tumors this changes the highly crosslinked and organized LAMA3B-rich EC-BM structure of the mammary gland (48) to a highly dynamic LAMA4-rich structure. Surprisingly, however, Lm322, which contains LAMA3B, is associated with aggressive breast cancers (49). The replacement of one vascular laminin for another is not a phenomenon limited to breast cancer; indeed, it occurs in many other solid tumors (50, 51). In many cases, the change involves the substitution of the β2 chain by the β1 chain. It is thought that β2 degradation by tumor-derived proteases triggers a compensatory increase in β1 expression. In oral squamous carcinoma cells, the switch from Lm511 to Lm411 is directed by snail, which leaves cancer cells with reduced adhesive capacity and therefore enhanced invasiveness (52). Snail also promotes epithelial-to-mesenchymal transition in these cancer cells. In addition to EC-BM modification, some pro-inflammatory cytokines—such as IL-1, IL-6, or TNF-α, cause alterations in many vasculature features, which in turn may affect immune function in tumors and other pathologies (53, 54).

Oxidation reactions lead to important modifications of the EC-BM in tumors (55), affecting the interaction of EC-BM proteins with cell components. For example, oxidized Lm111 increases monocyte adhesion to human umbilical vein endothelial cell (HUVEC) monolayers due to increased ICAM-1 expression (56). The increased attachment of EC to oxidized laminins might reflect an *in vivo* tendency for the quick coverage by EC of lesions exposed to oxidative stress. The influence of oxidative stress on tumor endothelium is, however, largely unknown, despite EC-BM modification being a common feature in tumor progression.

## Regulation of SOD3 Activity in Cancer

SOD3 is a metalloenzyme predominantly located in the extracellular space, either in a soluble state or associated with the ECM (57). In fibroblasts and ECs, SOD3 can also be found in the nucleus and cytoplasm (58, 59), although its function in these cell locations is unclear. The active homotetrameric form of SOD3 requires each monomer to complex with one zinc and two copper ions in the catalytic domain for $\cdot {O_{2}}^{-}$ anions to be efficiently dismutated (60).

SOD3 expression is basal in most tissues, but it is much higher in the lung, kidney, uterus, placenta, and throughout the cardiovascular system (61). Oncogenesis typically downregulates SOD3; indeed, this SOD isoform is that which shows the greatest difference in expression between tumors and healthy tissues (62). Nevertheless, the role of SOD3 in oncogenesis is unsure. In support of it having a pro-tumorigenic role, SOD3 levels correlate positively with the growth rate of some benign tumors, and high levels of the enzyme promote the growth and transformation of mouse embryonic fibroblasts. SOD3 also participates in vascular endothelial growth factor (VEGF)-C-driven breast tumorigenesis (63, 64). Many of these pro-tumorigenic activities have been linked to sustained mitogen-activated protein kinase pathway activation in preneoplastic cells *via* either the direct regulation of tyrosine kinase receptor phosphorylation, or small Rho/Ras GTPase signaling (64).

Interestingly, SOD3 negatively regulates other pro-oncogenic signaling pathways, such as those inducing the NF-κB and HIF-1α cascades (65). It also triggers DNA damage-induced apoptosis (66), which might explain the SOD3-induced inhibition of chemically induced melanomas (67). As indicated above, SOD3 is repressed in many solid tumors *via* epigenetic silencing (promoter hypermethylation and histone modification), messenger RNA (mRNA) targeting by oncomiR-21, and mutations in the promoter or the heparin-binding domain of the SOD3 gene (62, 68–70). The chromosomal region containing the SOD3 gene (4p15.1–4p15.3) is a hotspot for loss of heterozygosity (LOH) in cancer (62); an LOH rate of 30–60% is seen in tumors with low SOD3 levels (71, 72). The rate of allelic deletion increases drastically between cervical intraepithelial neoplasia and grade I cervical cancer, suggesting that SOD3 downregulation is associated with tumor progression.

SOD3 also has anti-tumor effects by altering the structure, composition, and dynamics of the extracellular matrix (62). Many of these effects are mediated *via* the antioxidative protection of the matrix from ROS/RNS-induced oxidative fragmentation. In other cases, SOD3 inhibits metalloproteinase activity or the expression of ECM-degrading enzymes such as heparanase (73). Heparanase promotes cancer growth, metastasis, and angiogenesis by inducing the degradation of heparan sulfate (HS), a sulfated glycosaminoglycan. SOD3 protects the integrity of HS by reducing heparanase expression and by preventing ROS-mediated HS degradation, thus reducing cancer cell proliferation and invasion (73). SOD3 might therefore act as a cancer cell-intrinsic and extrinsic tumor suppressor.

## SOD3 Effects on Tumor Vasculature

Vascular oxidative stress has broad effects on blood vessel function, and is central to the onset and progression of many cardiovascular diseases (74). ROS are also inducers of tumor angiogenesis *via* the generation of oxidized lipids and the induction of the HIF-1α/VEGF-A pathway in EC and VSMC (75). ROS regulates NO bioavailability through its oxidation to peroxynitrite. NO is a soluble intermediate messenger for EC and VSMC involved in angiogenesis, vascular leakage, and vascular tone maintenance (76–78). ROS also have a harmful influence on the EC inflammatory response. Together with reduced NO bioavailability and the imbalanced production of endothelium-derived relaxing and contractile factors, high ROS levels in tissues inexorably lead to EC dysfunction and vascular disorders (79–81).

By reducing ROS, extracellular (but not intracellular) SOD3 activity preserves endothelial integrity and function (82). By increasing perivascular NO bioavailability, SOD3 is vasorelaxing (83, 84). In cancer, SOD3 upregulation increases mean vessel area and tumor vessel length, although reduces vessel diameter. At ultrastructural level, SOD3 causes smoothness of the vessel lumen, consistent with enhanced EC quiescence, reduces vascular permeability, and improves tumor perfusion. These activities rely on the prevention of HIF-2α degradation in SOD3-overexpressing EC (13). The regulation of a transcription factor (HIF-2α) by an extracellular enzyme (SOD3) occurs through the increased levels of perivascular NO, a known inhibitor of the oxygen-sensing prolyl hydroxylases domain proteins (PHDs) that target HIF-2α for proteasome-mediated degradation. NO then fosters nuclear HIF-2α accumulation and the initiation of a transcriptional program that upregulates VEC and prevents AJ destabilization. EC-specific PHD2 hemizygosity also strengthens EC adhesion by increasing HIF-2α-dependent VEC transcription (85). SOD3 upregulation and PHD-2 reduction thus improve vascular function *via* the same pathway, which in turn enhances chemotherapeutic drug delivery and tumor shrinkage (13, 85). In primary colorectal cancers, it was found a significant correlation between enhanced SOD3 mRNA levels, stromal HIF-2α stabilization and VEC expression (13), suggesting that this SOD3-HIF-2α pathway also stabilizes the AJ in the vasculature of human tumors.

## SOD3 Regulation of the Inflammatory Response

In cancer immunotherapy it is important to determine the link between vascular alterations and T-cell exclusion from tumors. Preclinical evidence in breast, pancreatic and brain tumors, and in melanoma (4, 86), along with clinical trials in advanced kidney cancer (5), indicate that the combination of antiangiogenics and immune checkpoint inhibitor (ICI) increases TIL density and the antitumor immune responses. This synergistic effect might be a consequence of the vascular correction associated with antiangiogenics (mostly VEGF blockers) (87). Nevertheless, VEGF has direct immunosuppressive effects (3, 87), and therefore it is impossible to unequivocally link VEGF blockade-induced vascular normalization with TIL entry.

One of the tissue-protective activities of SOD3 is to downmodulate inflammation (88–90). SOD3 anti-inflammatory activity has been associated with the repression of adhesion receptors (VCAM-1, ICAM-1) and chemoattractants (CCL2, CXCL2) in EC (88). Indeed, SOD3 repression increases immune cell infiltration, particularly that of granulocytes, in the lung (91). In tumors, however, SOD3 shapes the inflammatory infiltrate in a complex manner, promoting the extravasation of specific leukocyte subtypes. The upregulation of SOD3 in the tumor stroma makes the tumor endothelium more permissive to adoptively transferred and endogenous tumor-specific CD4^+^ and CD8^+^ T-cells. However, the infiltration of myeloid and T_REG_ cells is unaffected (14). SOD3 thus creates an immunogenic TME that significantly reduces tumor growth and improves immunotherapy.

## SOD3-Induced WNT Pathway Activation, Endothelial Cell Basement Membrane Remodeling, and T-Cell Infiltration Into Tumors

SOD3 overexpression in EC monolayers fosters the transmigration of naive and activated T-cells (14). HIF-2α is again at the center of this SOD3 effect, influencing T-cell transmigration *in vitro* and TIL density *in vivo*. The mechanism by which the SOD3/HIF-2α pathway boosts T cell infiltration depends on autocrine WNT pathway activation. This is paradoxical since WNT signaling deregulation has been implicated in virtually all stages of oncogenesis, including transformation, metastatic dissemination, and resistance to immunotherapy (92, 93).

WNT signaling is initiated when a soluble WNT ligand binds to cognate receptors from the Frizzled (FZD) family (10 in human and mice). Depending on multiple parameters, including the precise identity of the ligand and the receptor, this interaction triggers different signal transduction cascades that culminate with the activation of transcriptional and post-translational programs (94). One of this cascades, the so-called “canonical,” relies on the cytosolic stabilization of β-catenin, which is usually targeted for proteosomal degradation by a supramolecular entity known as “β-catenin degradation complex,” build up by the tumor suppressor adenomatous polyposis coli (APC), axin-1, casein kinase-1α, and glycogen synthase kinase-3. Binding of the WNT ligand to the FZD in association with the co-receptor, low-density lipoprotein receptor-related protein-5 or -6, recruits disheveled segment polarity protein (DVL)-1 or -2, which bind to axin-1, thus preventing β-catenin degradation (92, 94).

The cytosolic accumulation of β-catenin, in conjunction with other DNA-binding proteins, triggers β-catenin accumulation into the nucleus where interacts with the T cell factor/lymphoid enhancer factor (TCF/LEF) family members (94). These complexes then initiates transcriptional programs implicated in cancer cell proliferation, stemness, polarity, metabolic reprogramming, and migration, among others. In addition, WNT signaling can occur in a β-catenin-independent manner, the so-called “non-canonical,” extensively reviewed elsewhere (95).

In addition to the cancer cell-autonomous effects, WNT pathway shapes vascular and immune functions in the tumor stroma. Myeloid cell-produced WNT ligands affect the angiogenic switch through a β-catenin-dependent EC metabolic reprograming to glycolysis (96, 97). WNT signaling also influences anti-tumor immune responses, although these effects are context-dependent. Tumor-intrinsic WNT signaling elements can be recognized by the immune system as tumor-associated antigens, serving as targets for the development of tumor-specific vaccines (98, 99). In contrast, β-catenin signaling in cancer cells drives primary immune resistance by skewing DC maturation to a tolerogenic phenotype (100), and by excluding effector T cells from the tumor microenvironment (92, 93, 101). In EC, however, WNT pathway activation transcriptionally upregulates LAMA4, a permissive signal for T-cell diapedesis (see above). LAMA4 levels correlate positively with TIL density after *SOD3* upregulation, whereas HIF-2α deletion or WNT pathway inhibition reduces LAMA4 and TIL density (14). These results suggest a signaling pathway connecting SOD3, HIF-2α, and WNT that culminates in transcriptional LAMA4 upregulation.

This SOD3/HIF-2α/LAMA4 pathway is operative in human primary colorectal cancer (CRC). SOD3 and LAMA4 levels correlate positively with enhanced TIL numbers in a small cohort of stage II CRC patients, and with the T-cell inflammatory signatures in the CRC cohort described in The Cancer Genome Atlas (14). In the stage II CRC cohort studied, SOD3 positivity was also associated with lower recurrence rates, which is consistent with the prognostic value of TIL density in CRC. However, the association of LAMA4 positivity with the relapse rate in the stage II cohort was less marked than that of SOD3. It may be that SOD3 not only enhances LAMA4 in EC-BM but also in the basement membrane underlying cancer cells. Since LAMA4 is associated with tumor aggressiveness (56), LAMA4 activity in cancer cells might counteract the anti-tumor effect associated with enhanced TIL entry.

## Concluding Remarks

Although ROS is a well-known inducer of vasculature malfunction in cardiovascular disease, its involvement in tumor vascular abnormalization is only vaguely recognized. Undoubtedly, however, oncogenesis brings forth a plethora of tissue and cell alterations leading to an excess of ROS in the TME. One of these alterations is the silencing of the anti-oxidant enzyme SOD3, which is a cornerstone of normal endothelial function. As reviewed here, SOD3 re-expression in the TME triggers a program that simultaneously induces vascular normalization and T-cell infiltration, unequivocally linking these processes. The SOD3 program operates in primary CRC, affecting the disease relapse rate and patient survival, an effect associated with increased intratumor CD8^+^ T-cell infiltration. As occurs in allograft transplants, LAMA4 works as a molecular switch that potentiates immunity over tolerance in the TME.

One of the most striking findings is that SOD3 induces the specific intra-tumor infiltration of effector T-cells. This was unexpected, since SOD3 acts as anti-inflammatory molecule in non-tumor models. The molecular explanation of this selective effect deserves further research. SOD3-induced LAMA4 upregulation would explain enhanced leukocyte extravasation but not the exclusive diapedesis of effector T-cells. SOD3 seems not to upregulate CXCR3 ligands, however, which are proposed to be a major chemoattractant for T-cell infiltration into tumors (2). A plausible explanation is that T-cell diapedesis specificity is achieved by the combination of different signals: the SOD3-induced upregulation of a permissive signal (LAMA4) for leukocyte extravasation, and an instructive signal for T-cell diapedesis induced by EC normalization. Whether this signaling network operates in metastatic disease deserves future research. It would also important to study how SOD3 affects blood vessels in tumor models in which SOD3 may function as pro-tumorigenic.

**Figure 2:**
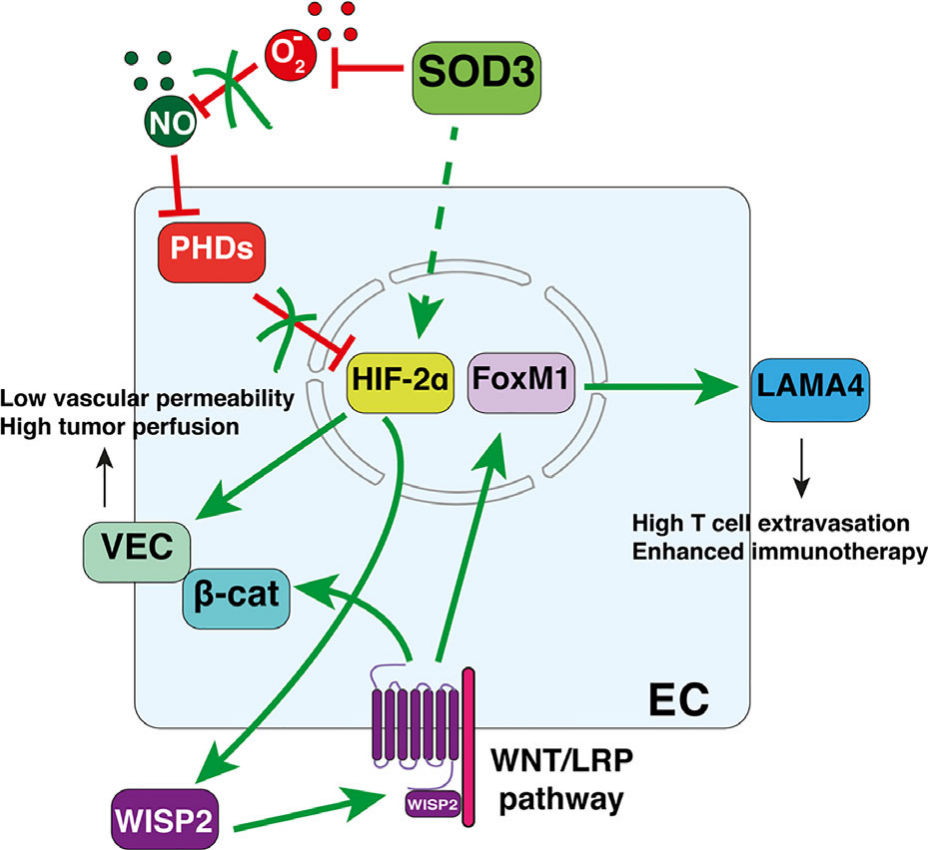
Superoxide dismutase-3 (SOD3)-induced pathways connecting vascular normalization and T-cell diapedesis in tumors. Perivascular SOD3 prevents nitric oxide (NO) oxidation, thus increasing EC NO levels. This causes PHD inhibition and the nuclear accumulation of HIF-2α. HIF-2α then initiates a transcriptional program that upregulates vascular-endothelial cadherin (VEC) and certain WNT ligands. Increased VEC levels reduce vascular permeability and normalize the vasculature. Autocrine WNT ligands activate the WNT pathway, which stabilizes β-catenin and FoxM1. β-catenin is sequestered in the juxtamembrane region by VEC, reinforcing AJs. FoxM1 translocates to the nucleus to induce LAMA4 transcription, providing a permissive signal for T-cell diapedesis.

Another open question is the role of the WNT pathway in TIL entry. WNT/β-catenin activation in neoplastic cells is a major T-cell exclusion pathway for different cancers (92, 93, 101). However, SOD3-induced T-cell extravasation relies on WNT pathway activation in EC (14). These contrasting tumor- and EC-specific effects of WNT on TIL entry would seem to reflect cell-type-specific outcomes of this signaling pathway. It should be noted that the WNT/β-catenin pathway only excludes T-cells from tumors when β-catenin translocates to the nucleus of cancer cells (102). SOD3-induced WNT pathway activation in EC, however, does not cause β-catenin to be imported into the nucleus since SOD3 also upregulates VEC, which sequesters β-catenin in the AJ (**Figure 2**). Indeed, SOD3-induced LAMA4 upregulation is dependent on FoxM1 (14), another WNT-stabilized transcription factor. The therapeutic implication of this divergence might be important since WNT/β-catenin inhibitors have not provided the expected clinical results. It is here proposed that it might be possible to design effective WNT-based immunotherapeutic strategies by targeting WNT/β-catenin inhibitors to specific tumor compartments.

## Author Contributions

All authors listed have made a substantial, direct, and intellectual contribution to the work and approved it for publication.

## Funding

This work was funded by the Spanish Ministry of Science, Innovation and Universities (SAF2017-83732-R; AEI/FEDER, EU), the *Comunidad de Madrid* (B2017/BMD-3733; Inmunothercan-CM), and the *Fundación Domingo Martínez* and the *Fundación Merck-Salud*. DM-R was supported by a predoctoral fellowship from the Spanish Ministry of Science, Innovation and Universities and the EU European Social Fund (PRE2018-084023).

## Conflict of Interest

The authors declare that the research was conducted in the absence of any commercial or financial relationships that could be construed as a potential conflict of interest.

## References

[1] Osipov A Saung MT Zheng L Murphy AG Small molecule immunomodulation: The tumor microenvironment and overcoming immune escape. J Immunother Cancer (2019) 7:224. 10.1186/s40425-019-0667-0 31439034 PMC6704558

[2] Lacalle RA Blanco R Carmona-Rodríguez L Martín-Leal A Mira E Mañes S Chemokine receptor signaling and the hallmarks of cancer. Int Rev Cell Mol Biol (2017) 331:181–244. 10.1016/bs.ircmb.2016.09.011 28325212

[3] Hilmi M Neuzillet C Calderaro J Lafdil F Pawlotsky JM Rousseau B Angiogenesis and immune checkpoint inhibitors as therapies for hepatocellular carcinoma: current knowledge and future research directions. J Immunother Cancer (2019) 7:333. 10.1186/s40425-019-0824-5 31783782 PMC6884868

[4] Schmittnaegel M Rigamonti N Kadioglu E Cassara A Wyser Rmili C Kiialainen A Dual angiopoietin-2 and VEGFA inhibition elicits antitumor immunity that is enhanced by PD-1 checkpoint blockade. Sci Transl Med (2017) 9:eaak9670. 10.1126/scitranslmed.aak9670 28404865

[5] Wallin JJ Bendell JC Funke R Sznol M Korski K Jones S Atezolizumab in combination with bevacizumab enhances antigen-specific T-cell migration in metastatic renal cell carcinoma. Nat Commun (2016) 7:12624. 10.1038/ncomms12624 27571927 PMC5013615

[6] Reuter S Gupta SC Chaturvedi MM Aggarwal BB Oxidative stress, inflammation, and cancer: How are they linked? Free Radic Biol Med (2010) 49:1603–16. 10.1016/j.freeradbiomed.2010.09.006 PMC2990475 20840865

[7] Sena CM Leandro A Azul L Seiça R Perry G Vascular Oxidative Stress: Impact and Therapeutic Approaches. Front Physiol (2018) 9:1668. 10.3389/fphys.2018.01668 30564132 PMC6288353

[8] Betteridge DJ What is oxidative stress? Metabolism (2000) 49:3–8. 10.1016/S0026-0495(00)80077-3 10693912

[9] Jones DP Redefining oxidative stress. Antioxid Redox Signal (2006) 8:1865–79. 10.1089/ars.2006.8.1865 16987039

[10] Bandyopadhyay U Das D Banerjee RK Reactive oxygen species: oxidative damage and pathogenesis. Curr Sci (1999) 77:658–66.

[11] Schieber M Chandel NS ROS function in redox signaling and oxidative stress. Curr Biol (2014) 24:R453–62. 10.1016/j.cub.2014.03.034 PMC4055301 24845678

[12] Santilli F D’Ardes D Davì G Oxidative stress in chronic vascular disease: From prediction to prevention. Vascul Pharmacol (2015) 74:23–37. 10.1016/j.vph.2015.09.003 26363473

[13] Mira E Carmona-Rodríguez L Pérez-Villamil B Casas J Fernández-Aceñero MJ Martínez-Rey D SOD3 improves the tumor response to chemotherapy by stabilizing endothelial HIF-2alpha. Nat Commun (2018) 9:575. 10.1038/s41467-018-03079-1 29422508 PMC5805714

[14] Carmona-Rodríguez L Martínez-Rey D Fernández-Aceñero MJ González-Martín A Paz-Cabezas M Rodríguez-Rodríguez N SOD3 induces a HIF-2α-dependent program in endothelial cells that provides a selective signal for tumor infiltration by T cells. J Immunother Cancer (2020) 8:e000432. 10.1136/jitc-2019-000432 32591431 PMC7319787

[15] Vestweber D How leukocytes cross the vascular endothelium. Nat Rev Immunol (2015) 15:692–704. 10.1038/nri3908 26471775

[16] Wimmer I Tietz S Nishihara H Deutsch U Sallusto F Gosselet F PECAM-1 stabilizes blood-brain barrier integrity and favors paracellular T-cell diapedesis across the blood-brain barrier during neuroinflammation. Front Immunol (2019) 10:711. 10.3389/fimmu.2019.00711 31024547 PMC6460670

[17] Schulte D Küppers V Dartsch N Broermann A Li H Zarbock A Stabilizing the VE-cadherinĝ€”catenin complex blocks leukocyte extravasation and vascular permeability. EMBO J (2011) 30:4157–70. 10.1038/emboj.2011.304 PMC3199392 21857650

[18] Muller WA Transendothelial migration: unifying principles from the endothelial perspective. Immunol Rev (2016) 273:61–75. 10.1111/imr.12443 27558328 PMC5090979

[19] Marchand M Monnot C Muller L Germain S Extracellular matrix scaffolding in angiogenesis and capillary homeostasis. Semin Cell Dev Biol (2019) 89:147–56. 10.1016/j.semcdb.2018.08.007 30165150

[20] Di Russo J Hannocks MJ Luik AL Song J Zhang X Yousif L Vascular laminins in physiology and pathology. Matrix Biol (2017) 57–58:140–8. 10.1016/j.matbio.2016.06.008 27378388

[21] Simon-Assmann P Orend G Mammadova-Bach E Spenlé C Lefebvre O Role of laminins in physiological and pathological angiogenesis. Int J Dev Biol (2011) 55:455–65. 10.1387/ijdb.103223ps 21858771

[22] Pozzi A Yurchenco PD Iozzo RV The nature and biology of basement membranes. Matrix Biol (2017) 57–58:1–11. 10.1016/j.matbio.2016.12.009 PMC5387862 28040522

[23] Bixel MG Li H Petri B Khandoga AG Khandoga A Zarbock A CD99 and CD99L2 act at the same site as, but independently of, PECAM-1 during leukocyte diapedesis. Blood (2010) 116:1172–84. 10.1182/blood-2009-12-256388 20479283

[24] Yousif LF Di Russo J Sorokin L Laminin isoforms in endothelial and perivascular basement membranes. Cell Adh Migr (2013) 7:101–10. 10.4161/cam.22680 PMC3544773 23263631

[25] Thyboll J Kortesmaa J Cao R Soininen R Wang L Iivanainen A Deletion of the laminin alpha4 chain leads to impaired microvessel maturation. Mol Cell Biol (2002) 22:1194–202. 10.1128/mcb.22.4.1194-1202.2002 PMC134646 11809810

[26] Bolcato-Bellemin AL Lefebvre O Arnold C Sorokin L Miner JH Kedinger M Laminin alpha5 chain is required for intestinal smooth muscle development. Dev Biol (2003) 260:376–90. 10.1016/s0012-1606(03)00254-9 12921739

[27] Miner JH Cunningham J Sanes JR Roles for laminin in embryogenesis: exencephaly, syndactyly, and placentopathy in mice lacking the laminin alpha5 chain. J Cell Biol (1998) 143:1713–23. 10.1083/jcb.143.6.1713 PMC2132973 9852162

[28] Song J Lokmic Z Lämmermann T Rolf J Wu C Zhang X Extracellular matrix of secondary lymphoid organs impacts on B-cell fate and survival. Proc Natl Acad Sci U S A (2013) 110:E2915–24. 10.1073/pnas.1218131110 PMC3732919 23847204

[29] Sixt M Engelhardt B Pausch F Hallmann R Wendler O Sorokin LM Endothelial cell laminin isoforms, laminins 8 and 10, play decisive roles in T cell recruitment across the blood-brain barrier in experimental autoimmune encephalomyelitis. J Cell Biol (2001) 153:933–46. 10.1083/jcb.153.5.933 PMC2174323 11381080

[30] Warren KJ Iwami D Harris DG Bromberg JS Burrell BE Laminins affect T cell trafficking and allograft fate. J Clin Invest (2014) 124:2204–18. 10.1172/JCI73683 PMC4001556 24691446

[31] Wu C Ivars F Anderson P Hallmann R Vestweber D Nilsson P Endothelial basement membrane laminin alpha5 selectively inhibits T lymphocyte extravasation into the brain. Nat Med (2009) 15:519–27. 10.1038/nm.1957 19396173

[32] Kenne E Soehnlein O Genove G Rotzius P Eriksson EE Lindbom L Immune cell recruitment to inflammatory loci is impaired in mice deficient in basement membrane protein laminin alpha4. J Leukoc Biol (2010) 88:523–8. 10.1189/jlb.0110043 20483922

[33] Zhang X Wang Y Song J Gerwien H Chuquisana O Chashchina A The endothelial basement membrane acts as a checkpoint for entry of pathogenic T cells into the brain. J Exp Med (2020) 217:e20191339. 10.1084/jem.20191339 32379272 PMC7336306

[34] Song J Zhang X Buscher K Wang Y Wang H Di Russo J Endothelial basement membrane laminin 511 contributes to endothelial junctional tightness and thereby inhibits leukocyte transmigration. Cell Rep (2017) 18:1256–69. 10.1016/j.celrep.2016.12.092 28147279

[35] Ochando JC Yopp AC Yang Y Garin A Li Y Boros P Lymph Node Occupancy Is Required for the Peripheral Development of Alloantigen-Specific Foxp3 + Regulatory T Cells. J Immunol (2005) 174:6993–7005. 10.4049/jimmunol.174.11.6993 15905542

[36] García-Nieto S Johal RK Shakesheff KM Emara M Royer PJ Chau DY Laminin and fibronectin treatment leads to generation of dendritic cells with superior endocytic capacity. PloS One (2010) 5:e10123. 10.1371/journal.pone.0010123 20419094 PMC2856673

[37] Geberhiwot T Assefa D Kortesmaa J Ingerpuu S Pedraza C Wondimu Z Laminin-8 (alpha4beta1gamma1) is synthesized by lymphoid cells, promotes lymphocyte migration and costimulates T cell proliferation. J Cell Sci (2001) 114:423–33. 10.1242/jcs.114.2.423 11148143

[38] Simon T Li L Wagner C Zhang T Saxena V Brinkman CC Differential Regulation of T-cell Immunity and Tolerance by Stromal Laminin Expressed in the Lymph Node. Transplantation (2019) 103:2075–89. 10.1097/TP.0000000000002774 PMC6768765 31343575

[39] Baluk P Morikawa S Haskell A Mancuso M McDonald DM Abnormalities of Basement Membrane on Blood Vessels and Endothelial Sprouts in Tumors. Am J Pathol (2003) 163:1801–15. 10.1016/s0002-9440(10)63540-7 PMC1892429 14578181

[40] Kalluri R Basement membranes: structure, assembly and role in tumour angiogenesis. Nat Rev Cancer (2003) 3:422–33. 10.1038/nrc1094 12778132

[41] Mira E Lacalle RA Buesa JM González de Buitrago G Jiménez-Baranda S Gómez-Moutón C Secreted MMP9 promotes angiogenesis more efficiently than constitutive active MMP9 bound to the tumor cell surface. J Cell Sci (2004) 117:1847–56. 10.1242/jcs.01035 15075244

[42] Avraamides CJ Garmy-Susini B Varner JA Integrins in angiogenesis and lymphangiogenesis. Nat Rev Cancer (2008) 8:604–17. 10.1038/nrc2353 PMC2577722 18497750

[43] Xu J Rodriguez D Petitclerc E Kim JJ Hangai M Moon YS Proteolytic exposure of a cryptic site within collagen type IV is required for angiogenesis and tumor growth in vivo. J Cell Biol (2001) 154:1069–79. 10.1083/jcb.200103111 PMC2196184 11535623

[44] Kim YM Jang JW Lee OH Yeon J Choi EY Kim KW Endostatin inhibits endothelial and tumor cellular invasion by blocking the activation and catalytic activity of matrix metalloproteinase. Cancer Res (2000) 60:5410–3. 11034081

[45] Colorado PC Torre A Kamphaus G Maeshima Y Hopfer H Takahashi K Anti-angiogenic cues from vascular basement membrane collagen. Cancer Res (2000) 60:2520–6. 10811134

[46] Trachana V Christophorides E Kouzi-Koliakos K Koliakos G Laminin-1 is phosphorylated by ecto-protein kinases of monocytes. Int J Biochem Cell Biol (2005) 37:478–92. 10.1016/j.biocel.2004.08.001 15474991

[47] Mori T Kariya Y Komiya E Higashi S Miyagi Y Sekiguchi K Downregulation of a newly identified laminin, laminin-3B11, in vascular basement membranes of invasive human breast cancers. Cancer Sci (2011) 102:1095–100. 10.1111/j.1349-7006.2011.01892.x 21276136

[48] Kariya Y Mori T Yasuda C Watanabe N Kaneko Y Nakashima Y Localization of laminin alpha3B chain in vascular and epithelial basement membranes of normal human tissues and its down-regulation in skin cancers. J Mol Histol (2008) 39:435–46. 10.1007/s10735-008-9183-0 18670895

[49] Carpenter PM Ziogas A Markham EM Cantillep AS Yan R Anton-Culver H Laminin 332 expression and prognosis in breast cancer. Hum Pathol (2018) 82:289–96. 10.1016/j.humpath.2018.08.003 PMC6289632 30125583

[50] Ljubimova JY Lakhter AJ Loksh A Yong WH Riedinger MS Miner JH Overexpression of alpha4 chain-containing laminins in human glial tumors identified by gene microarray analysis. Cancer Res (2001) 61:5601–10. 10.3892/ijo.18.2.287 11454714

[51] Fujita M Khazenzon NM Bose S Sekiguchi K Sasaki T Carter WG Overexpression of beta1-chain-containing laminins in capillary basement membranes of human breast cancer and its metastases. Breast Cancer Res (2005) 7:R411–21. 10.1186/bcr1011 PMC1175051 15987446

[52] Takkunen M Ainola M Vainionpää N Grenman R Patarroyo M García de Herreros A Epithelial-mesenchymal transition downregulates laminin alpha5 chain and upregulates laminin alpha4 chain in oral squamous carcinoma cells. Histochem Cell Biol (2008) 130:509–25. 10.1007/s00418-008-0443-6 18496706

[53] Aguilar-Cazares D Chavez-Dominguez R Carlos-Reyes A Lopez-Camarillo C Hernadez de la Cruz ON Lopez-Gonzalez JS Contribution of Angiogenesis to Inflammation and Cancer. Front Oncol (2019) 9:1399. 10.3389/fonc.2019.01399 31921656 PMC6920210

[54] Wolf D Zirlik A Ley K Beyond vascular inflammation - Recent advances in understanding atherosclerosis. Cell Mol Life Sci (2015) 72:3853–69. 10.1007/s00018-015-1971-6 PMC4577451 26100516

[55] Kersh EN Kaech SM Onami TM Moran M Wherry EJ Miceli MC TCR signal transduction in antigen-specific memory CD8 T cells. J Immunol (2003) 170:5455–63. 10.4049/jimmunol.170.11.5455 12759421

[56] Kostidou E Topouridou K Daniilidis A Kaloyianni M Koliakos G Oxidized laminin-1 induces increased monocyte attachment and expression of ICAM-1 in endothelial cells. Int J Exp Pathol (2009) 90:630–7. 10.1111/j.1365-2613.2009.00686.x PMC2803254 19958399

[57] Sandström J Nilsson P Karlsson K Marklund SL 10-Fold increase in human plasma extracellular superoxide dismutase content caused by a mutation in heparin-binding domain. J Biol Chem (1994) 269:19163–6. 8034674

[58] Chu Y Piper R Richardson S Watanabe Y Patel P Heistad DD Endocytosis of extracellular superoxide dismutase into endothelial cells: Role of the heparin-binding domain. Arterioscler Thromb Vasc Biol (2006) 26:1985–90. 10.1161/01.ATV.0000234921.88489.5c 16809550

[59] Ookawara T Kizaki T Takayama E Imazeki N Matsubara O Ikeda Y Nuclear translocation of extracellular superoxide dismutase. Biochem Biophys Res Commun (2002) 296:54–61. 10.1016/S0006-291X(02)00804-5 12147226

[60] Bae JY Koo BK Ryu HB Song JA Nguyen MT Vu TTT Cu/Zn incorporation during purification of soluble human EC-SOD from E. coli stabilizes proper disulfide bond formation. Appl Biochem Biotechnol (2013) 169:1633–47. 10.1007/s12010-012-0025-x 23329142

[61] Nozik-Grayck E Suliman HB Piantadosi CA Extracellular superoxide dismutase. Int J Biochem Cell Biol (2005) 37:2466–71. 10.1016/j.biocel.2005.06.012 16087389

[62] Griess B Tom E Domann F Teoh-Fitzgerald M Extracellular superoxide dismutase and its role in cancer. Free Radic Biol Med (2017) 112:464–79. 10.1016/j.freeradbiomed.2017.08.013 PMC5685559 28842347

[63] Wang CA Harrell JC Iwanaga R Jedlicka P Ford HL Vascular endothelial growth factor C promotes breast cancer progression via a novel antioxidant mechanism that involves regulation of superoxide dismutase 3. Breast Cancer Res (2014) 16:462. 10.1186/s13058-014-0462-2 25358638 PMC4303136

[64] Laukkanen MO Extracellular Superoxide Dismutase: Growth Promoter or Tumor Suppressor? Oxid Med Cell Longev (2016) 2016:15–23. 10.1155/2016/3612589 PMC4880707 27293512

[65] Kim Y Kim BH Lee H Jeon B Lee YS Kwon MJ Regulation of skin inflammation and angiogenesis by EC-SOD via HIF-1α and NF-κB pathways. Free Radic Biol Med (2011) 51:1985–95. 10.1016/j.freeradbiomed.2011.08.027 21925591

[66] Kim J Mizokami A Shin M Izumi K Konaka H Kadono Y SOD3 acts as a tumor suppressor in PC-3 prostate cancer cells via hydrogen peroxide accumulation. Anticancer Res (2014) 34:2821–32. 24922645

[67] Kim S-H Kim M-O Gao P Youm C-A Park H-R Lee S-R Overexpression of extracellular superoxide dismutase (EC-SOD) in mouse skin plays a protective role in DMBA/TPA-induced tumor formation. Oncol Res (2005) 15:333–41. 10.3727/096504005776449725 16491951

[68] Teoh-Fitzgerald ML Fitzgerald MP Zhong W Askeland RW Domann FE Epigenetic reprogramming governs EcSOD expression during human mammary epithelial cell differentiation, tumorigenesis and metastasis. Oncogene (2014) 33:358–68. 10.1038/onc.2012.582 PMC3711965 23318435

[69] Teoh-Fitzgerald ML Fitzgerald MP Jensen TJ Futscher BW Domann FE Genetic and epigenetic inactivation of extracellular superoxide dismutase promotes an invasive phenotype in human lung cancer by disrupting ECM homeostasis. Mol Cancer Res (2012) 10:40–51. 10.1158/1541-7786.MCR-11-0501 22064654 PMC3262094

[70] Zhang X Ng WL Wang P Tian LL Werner E Wang H MicroRNA-21 modulates the levels of reactive oxygen species by targeting SOD3 and TNFα. Cancer Res (2012) 72:4707–13. 10.1158/0008-5472.CAN-12-0639 PMC3445705 22836756

[71] Polascik TJ Chang WYH Schoenberg MP Chang WYH Cairns P Sidransky D Distinct Regions of Allelic Loss on Chromosome 4 in Human Primary Bladder Carcinoma. Cancer Res (1995) 55:5396–9. 7585608

[72] Shivapurkar N Virmani AK Wistuba II Milchgrub S Mackay B Minna JD Deletions of chromosome 4 at multiple sites are frequent in malignant mesothelioma and small cell lung carcinoma. Clin Cancer Res (1999) 5:17–23. 9918198

[73] Teoh MLT Fitzgerald MP Oberley LW Domann FE Overexpression of extracellular superoxide dismutase attenuates heparanase expression and inhibits breast carcinoma cell growth and invasion. Cancer Res (2009) 69:6355–63. 10.1158/0008-5472.CAN-09-1195 PMC2728021 19602586

[74] Fukai T Folz RJ Landmesser U Harrison DG Extracellular superoxide dismutase and cardiovascular disease. Cardiovasc Res (2002) 55:239–49. 10.1016/S0008-6363(02)00328-0 12123763

[75] Kim YW Byzova TV Oxidative stress in angiogenesis and vascular disease. Blood (2014) 123:625–31. 10.1182/blood-2013-09-512749 PMC3907751 24300855

[76] Zhao Y Vanhoutte PM Leung SWS Vascular nitric oxide: Beyond eNOS. J Pharmacol Sci (2015) 129:83–94. 10.1016/j.jphs.2015.09.002 26499181

[77] Ziche M Morbidelli L Nitric oxide and angiogenesis. J Neurooncol (2000) 50:139–48. 10.1023/A:1006431309841 11245273

[78] Durán WN Beuve AV Sánchez FA Nitric oxide, S-Nitrosation, and endothelial permeability. IUBMB Life (2013) 65:819–26. 10.1002/iub.1204 PMC4004350 24078390

[79] Craige SM Kant S Keaney JF Reactive Oxygen Species in Endothelial Function: From Disease to Adaptation. Circ J (2015) 79:1145–55. 10.1253/circj.CJ-15-0464 25986771

[80] Endemann DH Schiffrin EL Endothelial dysfunction. J Am Soc Nephrol (2004) 15:1983–92. 10.1097/01.ASN.0000132474.50966.DA 15284284

[81] Incalza MA D’Oria R Natalicchio A Perrini S Laviola L Giorgino F Oxidative stress and reactive oxygen species in endothelial dysfunction associated with cardiovascular and metabolic diseases. Vascul Pharmacol (2018) 100:1–19. 10.1016/j.vph.2017.05.005 28579545

[82] Foresman EL Miller FJ Extracellular but not cytosolic superoxide dismutase protects against oxidant-mediated endothelial dysfunction. Redox Biol (2013) 1:292–6. 10.1016/j.redox.2013.04.003 PMC3757697 24024163

[83] Demchenko IT Gutsaeva DR Moskvin AN Zhilyaev SY Involvement of extracellular superoxide dismutase in regulating brain blood flow. Neurosci Behav Physiol (2010) 40:173–8. 10.1007/s11055-009-9240-5 20033309

[84] Lob HE Vinh A Li L Blinder Y Offermanns S Harrison DG Role of vascular extracellular superoxide dismutase in hypertension. Hypertension (2011) 58:232–9. 10.1161/HYPERTENSIONAHA.111.172718 PMC3376381 21730294

[85] Mazzone M Dettori D de Oliveira RL Loges S Schmidt T Jonckx B Heterozygous deficiency of PHD2 restores tumor oxygenation and inhibits metastasis via endothelial normalization. Cell (2009) 136:839–51. 10.1016/j.cell.2009.01.020 PMC4037868 19217150

[86] Allen E Jabouille A Rivera LB Lodewijckx I Missiaen R Steri V Combined antiangiogenic and anti-PD-L1 therapy stimulates tumor immunity through HEV formation. Sci Transl Med (2017) 9:eaak9679. 10.1126/scitranslmed.aak9679 28404866 PMC5554432

[87] Fukumura D Kloepper J Amoozgar Z Duda DG Jain RK Enhancing cancer immunotherapy using antiangiogenics: opportunities and challenges. Nat Rev Clin Oncol (2018) 15:325–40. 10.1038/nrclinonc.2018.29 PMC5921900 29508855

[88] Laurila JP Laatikainen LE Castellone MD Laukkanen MO SOD3 reduces inflammatory cell migration by regulating adhesion molecule and cytokine expression. PloS One (2009) 4:e5786. 10.1371/journal.pone.0005786 19495415 PMC2686160

[89] Kwon MJ Kim BH Lee YS Kim TY Role of superoxide dismutase 3 in skin inflammation. J Dermatol Sci (2012) 67:81–7. 10.1016/j.jdermsci.2012.06.003 22763488

[90] Sah SK Agrahari G Kim TY Insights into superoxide dismutase 3 in regulating biological and functional properties of mesenchymal stem cells. Cell Biosci (2020) 10:1–12. 10.1186/s13578-020-00386-3 32128111 PMC7045732

[91] Gongora MC Lob HE Landmesser U Guzik TJ Martin WD Ozumi K Loss of extracellular superoxide dismutase leads to acute lung damage in the presence of ambient air: a potential mechanism underlying adult respiratory distress syndrome. Am J Pathol (2008) 173:915–26. 10.2353/ajpath.2008.080119 PMC2543061 18787098

[92] Galluzzi L Spranger S Fuchs E Lopez-Soto A WNT signaling in cancer immunosurveillance. Trends Cell Biol (2019) 29:44–65. 10.1016/j.tcb.2018.08.005 30220580 PMC7001864

[93] Suryawanshi A Hussein MS Prasad PD Manicassamy S Wnt Signaling Cascade in Dendritic Cells and Regulation of Anti-tumor Immunity. Front Immunol (2020) 11:122. 10.3389/fimmu.2020.00122 32132993 PMC7039855

[94] Nusse R Clevers H Wnt/beta-catenin signaling, disease, and emerging therapeutic modalities. Cell (2017) 169:985–99. 10.1016/j.cell.2017.05.016 28575679

[95] Yang Y Mlodzik M Wnt-Frizzled/Planar Cell Polarity Signaling: Cellular Orientation by Facing the Wind (Wnt). Annu Rev Cell Dev Biol (2015) 31:623–46. 10.1146/annurev-cellbio-100814-125315 PMC4673888 26566118

[96] Yeo EJ Cassetta L Qian BZ Lewkowich I Li JF Stefater JA Myeloid wnt7b mediates the angiogenic switch and metastasis in breast cancer. Cancer Res (2014) 74:2962–73. 10.1158/0008-5472.CAN-13-2421 PMC4137408 24638982

[97] Pate KT Stringari C Sprowl-Tanio S Wang K TeSlaa T Hoverter NP Wnt signaling directs a metabolic program of glycolysis and angiogenesis in colon cancer. EMBO J (2014) 33:1454–73. 10.15252/embj.201488598 PMC4194089 24825347

[98] Robbins PF El-Gamil M Li YF Kawakami Y Loftus D Appella E A mutated β-catenin gene encodes a melanoma-specific antigen recognized by tumor infiltrating lymphocytes. J Exp Med (1996) 183:1185–92. 10.1084/jem.183.3.1185 PMC2192326 8642260

[99] Qian J Zheng Y Zheng C Wang L Qin H Hong S Active vaccination with Dickkopf-1 induces protective and therapeutic antitumor immunity in murine multiple myeloma. Blood (2012) 119:161–9. 10.1182/blood-2011-07-368472 PMC3251227 22049519

[100] Yaguchi T Goto Y Kido K Mochimaru H Sakurai T Tsukamoto N Immune suppression and resistance mediated by constitutive activation of Wnt/β-Catenin signaling in human melanoma cells. J Immunol (2012) 189:2110–7. 10.4049/jimmunol.1102282 22815287

[101] Luke JJ Bao R Sweis RF Spranger S Gajewski TF WNT/beta-catenin pathway activation correlates with immune exclusion across human cancers. Clin Cancer Res (2019) 25:3074–83. 10.1158/1078-0432.CCR-18-1942 PMC6522301 30635339

[102] Grasso CS Giannakis M Wells DK Hamada T Mu XJ Quist M Genetic mechanisms of immune evasion in colorectal cancer. Cancer Discov (2018) 8:730–49. 10.1158/2159-8290.CD-17-1327 PMC5984687 29510987

